# The phenotype and genotype of rheumatoid arthritis in the Democratic Republic of Congo

**DOI:** 10.1186/ar4269

**Published:** 2013-08-19

**Authors:** JJ Malemba, JM Mbuyi-Muamba, J Mukaya, X Bossuyt, MP Emonds, K Deiteren, R Westhovens, P Verschueren

**Affiliations:** 1 Department of Internal Medicine, Unit of Rheumatology, Kinshasa University Hospital, Kinshasa XI, Democratic Republic of Congo; 2 Unit of Radiology, Kinshasa University Hospital, Kinshasa XI, Democratic Republic of Congo; 3 Experimental Laboratory Immunology, Department of Medical Diagnostic Sciences, KU Leuven, Belgium; 4 Histocompatibility and Immunogenetics Laboratory (HILA), Red Cross Flanders, Mechelen, Belgium; 5 Division of Rheumatology, Department of Development and Regeneration, Neuromusculo-Skeletal Research Unit, KU Leuven, Belgium

**Keywords:** rheumatoid arthritis, phenotype, HLA-DRB1 genotype, DR Congo, Africa

## Abstract

**Introduction:**

Little is known about rheumatoid arthritis in the black, particularly in Congolese, populations. Our objective was to describe the phenotype and genotype of rheumatoid arthritis (RA) in Congolese.

**Methods:**

All consecutive rheumatoid arthritis (RA) patients attending Kinshasa University Hospital in a three-year time period were included. Demographics, clinical features and tobacco consumption were noted. Disease Activity Score (DAS)-28 based on the erythrocyte sedimentation rate (ESR), Health Assessment Questionnaire (HAQ), anti-citrullinated peptide antibodies (CCP) antibodies and rheumatoid factor (RF) were determined. Radiographs were scored according to Sharp-van der Heijde. On a subset of patients and controls HLA-DRB1 typing was performed.

**Results:**

A total of 114 females and 14 males aged 51.2 ± 14.9 were included. Mean duration of symptoms was four years. Moderate tobacco consumption was reported in a minority of patients. DAS-28 at first visit was >5.1 and HAQ ≥0.5 in all patients. X-rays showed joint erosions and/or joint space narrowing, mostly of a moderate grade in 55.8% of patients. Anti-CCP and/or RF were present in 48.6% of patients with available data (*n *= 72) and in 3.0% of controls (*n *= 67). Radiographic changes and nodules were more frequent in RF or anti-CCP positive patients. One copy of the shared epitope was found in 13 patients (35.1%) and 3 controls (12.5%). Two copies were found in one patient (2.7%) and in one control (4.2%).

**Conclusion:**

Congolese patients with RA consult long after disease onset. Despite this delay, the majority presents without major damage and is RF, anti-CCP and SE negative. We put forward the hypothesis that besides different environmental factors there is probably also a particular genetic risk profile in Congolese patients, different from the HLA-DRB1 shared epitope.

## Introduction

Rheumatoid arthritis (RA) is a systemic inflammatory disease characterized by symmetric destructive arthritis that is often associated with extra-articular and systemic manifestations. We must also take into account an important morbidity and socio-economic impact [1,2] of this disease as well as an increased cardio-vascular mortality [3-6]. Great strides have been made on RA in recent years, including a better understanding of the genetic aspects, environmental factors and the disease's pathogenesis, at least in the Western world. These developments also concern the clinical approach, including methods used to assess the impact of RA as well as treatment principles, such as the early intensive therapy and treat-to-target approach, and the use of biologicals. In contrast, there are still few data on RA in many other parts of the world. Little is known about the epidemiology of RA in black Africa, its severity, the cross-cultural validity of the different classification criteria and measurement scores, the socio-economic aspects as well as the genetic and/or environmental factors leading to the disease. Therefore, just copying Western principles might not be correct.

In the Democratic Republic of Congo very few studies have been published on RA. In 2008, JJ Malemba and JM Mbuyi-Muamba reported, in a retrospective study, that RA was rare and mild in its clinical presentation at the University Hospital of Kinshasa (UHK) [7]. These observations were in agreement with those reported by Bwanahali *et al*. in 1995 [8]. But these studies did not use "quantifiable" parameters to assess the severity of RA, and the first mentioned study had a hiatus due to its retrospective nature. In addition, no information was given on the frequency of shared epitope (SE), smoking and the possible influence of a tropical environment with its specific background of infections. In a recent epidemiological study [9] also, a rather mild phenotype was suggested. The current study was initiated to describe prospectively the phenotype and HLA-DRB1 genotype of newly diagnosed RA patients attending the rheumatology unit of the UHK in order to assess the severity of the disease in this part of the world and to test the potential involvement of amino acids 70 to 74 in the third hypervariable region of HLA-DRB1 (SE).

## Materials and methods

### Patient characteristics

This study includes all patients who were received for the first time in the rheumatology unit of the UHK in the period from 1 January 2008 to 31 December 2010, and who fulfilled the 1987 American College of Rheumatology (ACR) classification criteria for RA [10]. The approval of the ethics committee of the University of Kinshasa and the consent of patients and controls were obtained. The UHK receives approximately 500 patients with rheumatic diseases (new cases) per year. Osteoarthritis is diagnosed in more than 50% of patients, systemic diseases, spondylarthropathies and crystal arthritis in approximately 25% of patients, soft tissue rheumatism in 16%, osteoporosis and septic arthritis are less frequent. The control group consisted of patients suffering from other rheumatic diseases (osteoarthritis, soft tissue rheumatism, osteoporosis): 59 women and 8 men aged between 18 and 74 years. The clinical evaluation involved general aspects (fever, weight loss), signs and localization of synovitis (small joints/large joints, symmetric or not) and the duration of morning stiffness. Typical joint deformities of RA were listed. Extra-articular manifestations were also checked, including rheumatoid nodules, sicca syndrome and symptoms of systemic manifestations like pericarditis and lung or pleural involvement. Tobacco consumption was registered. At the initial visit and before prescribing anti-rheumatic drugs, the Disease Activity Score (DAS-28) was calculated [11] as well as the Health Assessment Questionnaire (HAQ index) [12] to assess functional impairment. The DAS-28 calculation was based on the erythrocyte sedimentation rate after one hour. Items of the HAQ were explained and translated by the investigator whenever necessary. Radiographs of hands and feet in frontal plane were performed to evaluate joint space narrowing and erosions according to the Sharp score modified by van der Heijde [13].

### Detection of anti-citrullinated peptide antibodies and rheumatoid factor

#### Patient characteristics

Blood samples were taken from a subset of the patients (*n *= 72) before the start of anti-rheumatic drugs and from controls (*n *= 67).

The samples were centrifuged at the time of collection and serum was stored at -77°C. The anti-citrullinated peptide antibodies (anti-CCP) were measured by fluoroenzyme immunoassay using reagents from the second generation (Thermo Fisher, Freiburg, Germany) on an Immuno CAP 250 instrument (Thermo Fisher). Rheumatoid factor (RF) was measured by nephelometry using an Immage 800 instrument (Beckman-Coulter, Brea, CA, USA) using reagents from Beckman-Coulter. Cut-off values for European individuals were applied.

#### Hemoglobin concentration measurement

The hemoglobin was measured in patients and controls by spectrophotometry using a molecular absorption spectrophotometer (SP 2100 Spectrum, CHINCAN; Shanghai, China).

#### HLA-DRB1 genotyping and allele classification

HLA-DRB1 typing was performed on a subset of patients (*n *= 37) and a control group (*n *= 24). Genomic DNA was extracted from EDTA peripheral blood samples with the QIAsymphony platform and the DNA Mini or Midi Kits (Qiagen, Hilden, Germany). HLA-DRB1 typing was performed using the sequence specific oligo (SSO) PCR technique (Lifecodes HLA-DRB1 typing kit, Gen-Probe, San Diego, CA, USA) with locus specific exon 2 amplification and hybridization on colored microbeads (Luminex, Austin, TX, USA). Alternatively a sequence specific primer (SSP) PCR method was used (HLA-DR low, Orelup, Vienna, Austria). Polymorphisms located in exon 3 were not resolved. The presence of shared epitope alleles was determined for patients and controls and the HLA-DRB1 alleles were classified according to du Montcel *et al. *[14]. This classification takes into account the amino acids 70 to 74 of HLA-DRB1. The highest risk for Caucasians was shown for the S2/S3P combination followed by S2/S2, S3P/S3P, S2L, S3P/L, L/L (in decreasing risk order). L is a combination group comprising the low risk groups S3D, S1 and X.

### Statistical analysis

A Student's *t*-test was used to compare data in patients with and without available data for X-ray damage, RF or anti-CCP. The odds ratios (OR) and the 95% confidence intervals were calculated by logistic regression. Tests for association of contingency tables were performed using the two-tailed Fisher's exact test. A *P-*value <0.05 was considered statistically significant.

## Results

One hundred twenty-eight patients were included, 114 women and 14 men (sex ratio F/M of 8:1). This study population represents 8.5% of all new patients attending the rheumatology unit of the UHK in the three-year study period. The clinical characteristics of the patients are described in Table 1. The mean age of the patients was 51 ± 14.9 years (range 18 to 80 years). The average time between the onset of the symptoms and the first visit was four years (range two months to nine years), and the median delay was three years. Cigarette smoking was reported in a minority of patients (two males). Nine ladies reported to "snuff" tobacco powder through the nose. The DAS-28 at the first consultation was over 5.1 in all patients. A HAQ index >0.5 was reported in all but one patient. The average hemoglobin was 11.5 ± 0.6 g/dl for patients and 12.1 ± 0.6/dl for the control group; 31 patients (24.2%) had a hemoglobin concentration ≤10 g/dl. Rheumatoid nodules and typical joint deformities were found in 17 (13.3%) and 28 patients (21.8%), respectively, interosseous muscle atrophy in 20 patients (15.6%), and sicca syndrome in 2 patients (1.6%). Weight loss was mentioned by 60 patients (46.9%).

**Table 1:** Clinical characteristics of patients with X-ray and/or serology versus others

Patients	Average age (years)	Disease duration	DAS-28 >5.1	Clinical presentation*	Frequency of deformities
Without X-ray	53.3 ± 16.0	4.5 ± 2.4	100%	100%	21.6%
With X-ray	49.9 ± 14.0	3.5 ± 2.6	100%	100%	22.0%
Without serology	50.9 ± 15.3	4.1 ± 2.8	100%	100%	21.4%
With serology	51.6 ± 14.9	3.9 ± 2.9	100%	100%	22.2%

(*): symmetric arthritis, hand joints involvement and involvement ≥3 joint areas. DAS-28, Disease Activity Score 28

Radiographs of hands and feet were obtained in a subgroup of patients only (*n *= 78). Forty-three (55.1%) showed erosions and/or joint space narrowing. The average and median (P25, P75) values of the van der Heijde score were 17.6 and 2.5 (0, 13) respectively. A very high score (>100) was found in four patients. Anti-CCP antibodies and RF were measured in 72 patients and 67 controls. Anti-CCP antibodies were positive in 34 patients (47.2%), RF in 25 patients (34.7%), and 2 controls were anti-CCP+/RF+ (Table 2). Erosions and/or joint space narrowing were observed in 63% of anti-CCP positive patients whose X-ray of hands and feet were available, and in 54.3% of anti-CCP negative patients. A total of 11/13 patients with rheumatoid nodules had anti-CCP antibodies with very high titers (>300 U/l).

**Table 2:** Test performance characteristics of SE, anti-CCP and RF for RA association

Parameter		Patients % (n)	Controls % (n)	PPV	NPV	Specificity	sensitivity	OR	95% CI	*P-*value
**SE**	positive	35.1 (13)	12.5 (3)	81	47	87	35	3.8	0.9 to 15.1	0.07
	negative	64.9 (24)	87.5 (21)							
**RF**	positive	34.7 (25)	3.0 (2)	93	58	97	35	17.3	3.9 to 76.6	<0.0001
	negative	65.2 (47)	97.0 (65)							
**anti-CCP**	positive	47.2 (34)	3.0 (2)	94	63	97	47	29.1	6.6 to 127.9	<0.0001
	negative	52.7 (38)	97.0 (65)							

Anti-CCP, anti-cyclic citrullinated peptide; NPV, negative predictive value; PPV, positive predictive value; RA, rheumatoid arthritis; RF, rheumatoid factor; SE, shared epitope

Table 1 shows that the patients whose serologic and/or radiographic data were available were similar to those for whom these data were not obtained. All had symmetric arthritis, arthritis of hand joints and involvement of at least three joint areas. No statistically significant difference was observed between the average age of patients whose X-rays were available compared to patients without X-rays (*P *= 0.21) or between patients with serological analyzes and patients without (*P *= 0.74).

HLA-DRB1 typing was performed in a subset of patients (*n *= 37) and controls (*n *= 24). The most frequent HLA-DRB1 alleles in the control group were HLA-DRB1*15:03, 13:01/02, 03:01/02 and 11:01. There was a very low carrier frequency of DRB1*04:01 and DRB1*13:03 in our Congolese population (patient group and controls). The SE alleles detected were DRB1*04:01, *13:03, *01:01/02 and *10. One copy of the SE was found in 13 patients (35.1%) and 3 controls (12.5%). Two copies of the SE were found in 1 patient (2.7%) and 1 control (4.2%).

The association of the anti-CCP, RF and HLA-DRB1 SE test with RA was calculated, as shown in Table 2. The RF and anti-CCP tests have a high specificity (true negatives) and positive predictive value (PPV) with an OR = 17. 3 (*P *<0.0001) and OR = 29.1 (*P *<0.0001), respectively. However, the sensitivity and negative predictive value of these DNA tests are significantly lower. Although the HLA-DRB1 SE test had a specificity of 87% and a PPV of 81%, the OR was only 3.8 (95% CI 0.9 to 15.1) (*P *= 0.07). When the carrier status was counted, the OR was 2.6 (95% CI 0.8 to 8.3) (*P *= 0.124). The classification of HLA-DRB1 alleles according to du Montcel *et al*. showed the rarity of S2 HLA-DRB1 alleles (Table 3). We also looked into the association between the presence of the SE together with anti-CCP antibodies or RF. Using an univariate regression analysis, the association between SE and RF or SE and anti-CCP antibodies could not be established (*P *>0.05) (Table 4).

**Table 3:** HLA-DRB1 alleles classified according to du Montcel *et al*.

du Montcel classification	Amino acid sequence positions 70 to 74	HLA-DRB1	Patient group % (n)	Controls % (n)	SE	OR	95% CI	*P-v*alue
**S2**	QKRAA	*04:01	4.1 (3)	4.2 (2)	positive	2.6	0.8 to 8.3	0.124
	DKRAA	*13:03						
**S3P**	QRRAA	*01:01/02	14.9 (11)	4.2 (2)				
	RRRAA	*10						
**S1**	DERAA	*11:02, *13:01/02	81.0 (60)	91.6 (44)	negative			
	QARAA	*15						
**S3D**	DRRAA	*11:01, *12, *16						
**X**	nonXXRAA	*03,*07,*08,*09,*14						

[14], in RA patients and controls (homozygous alleles are counted double)

OR, odds ratio; RA, rheumatoid arthritis; SE, shared epitope; A, alanin; D, aspartic acid aspartique; E, glutamic acid; K, leucin; Q, glutamin; R, arginin

**Table 4:** Relation between shared epitope, anti-CCP and RF in Congolese RA patients

		**RA patient group**		**OR**	**95% CI**	***P-*value**
			
		SE positive	SE negative			
**anti-CCP**	positive	7	1	1.6	0.35 to 7.11	0.712
	negative	4	10			
**RF**	positive	4	9	0.76	0.17 to 3.4	1.0
	negative	7	12			

Anti-CCP, anti-cyclic citrullinated peptide; RA, rheumatoid arthritis; RF, rheumatoid factor

## Discussion

Our results indicate that in a three-year time period 8.5% of outpatients suffering from rheumatic diseases and attending the rheumatology unit of the UHK were diagnosed with RA. The relative RA frequency of 8.5% observed in this study is higher than that reported in a previous paper showing RA to affect 3.6% of the patients attending the rheumatology unit of UHK from 1988 to 2002 [7]. This is probably because of the retrospective nature of the latter study.

Patients consulted following a rather long delay after experiencing typical symptoms and all presented with a high value of DAS-28. This may be attributed to several factors: the poverty of the population with difficulties in accessing and paying for healthcare, the use of traditional medicine, and lack of information and education. It is, therefore, understandable why many patients consult only when symptoms become intolerable as a consequence of high disease activity. The high DAS-28-ESR scores in our patient population are probably partly related to the low hemoglobin level found in this study. These low hemoglobin levels may not necessarily be related to the severity of the RA since they are similar in patients and controls. They are probably influenced by the nutritional deficit and tropical infections which affect a part of the Congolese population. Therefore, it is probably unwise to compare DAS-28-ESR scores from Congolese patients in absolute terms with scores obtained in a Western population. We cannot exclude that DAS-28-CRP would have allowed a more accurate interpretation of the disease activity, but unfortunately, for practical reasons, CRP could not be determined.

Mean HAQ index was rather high, probably related to the disease activity, but rheumatoid nodules and typical deformities were found in less than 20% of patients. Erosions and joint space narrowing were found in somewhat more than 50% of patients and were of low to moderate severity, taking into account the high disease activity and important delay before treatment.

The fact that more than 50% of Congolese RA patients are RF and anti-CCP negative may contribute to the mildness of the disease phenotype since the level of anti-CCP and RF titers is linked with the disease severity [15-17]. A mild phenotype was also reported in other African populations. Adewolo *et al. *[18] observed that in 200 patients followed for RA in Nigeria only 38.5% were RF positive and 29% had joint erosions on radiographs of hands, while the average duration of symptoms was 63 months before the first consultation. Ndongo S *et al. *[19] in Senegal reported that joint erosions were present in one-third of patients and that extra-articular manifestations were very rare, while the average time from the onset of symptoms to initiation of disease modifying antirheumatic drugs was 54 months. Anaya JM *et al. *[20] in Colombia have described that RA was less severe in blacks than Mestizos in terms of radiographic damage. Recently, Singwe-Ngandeu *et al*. in Cameroon have also reported a mild presentation of RA among their patients [21].

RF and anti-CCP were observed in only a few controls (2.98%) in our study, whereas in this population, higher numbers were expected due to the high frequency of parasitic and viral infections. Using European cut-off values for RF and anti-CCP antibodies, as done in this study, may be a problem in a sub-Saharan African population and for future studies it would be advisable to determine local cut-off values.

In our study there does not seem to be an association between the presence of the shared epitope and RF or anti-CCP antibodies (Table 4). Classic HLA-DRB1 S2 and S3P epitopes were very rare. Singwe-Ngandeu M *et al. *[21] observed also a discrepancy in a group of RA patients in Cameroon of whom more than 75% were RF positive and anti-CCP positive, but only 30% were carriers of the S2 and S3P HLA-DRB1 alleles. The four most frequent HLA-DRB1 alleles in our control group are those typically reported in sub-Saharan Africa [22].

Interestingly, there was a low prevalence of HLA-DRB1*04:01 in this study population (patients and controls) compared to other populations [23,24]. This observation is in agreement with that of Mbayo *et al. *[25,26], who reported that HLA-DR4 was less frequent in Congolese blood donors. This may be important since some authors have reported that HLA-DRB1*04:01 (S2) is associated with the most severe phenotype of RA [23,27]. Other HLA-DRB1 alleles, on the contrary, were more prevalent. In agreement with the study of Mbayo [25,26], we observed a high frequency of HLA-DRB1*15: 03 (X), DRB1*13:01/02 (S1) and DRB1*11:01 (S3D). The lower risk of these HLA-DRB1 alleles is confirmed in different studies [28-34].

The present study showed that 35.1% of the study RA population had S2 and S3P HLA-DRB1 alleles. However, compared to the control group, the association of SE with RA was statistically not significant. Singwe-Ngandeu *et al*. in Cameroon observed a lower frequency (30%) of SE-positive cases among RA patients [21]. A low frequency of HLA-DRB1 SE in blacks was also observed by LB Hughes *et al. *[35], who reported a frequency of 25.2% in African American patients. They found a high degree of European ancestry among African Americans with SE alleles, which suggests that a genetic risk factor for RA was introduced into the African American population through admixture. This is not in agreement with the study of PWA Meyer *et al. *[36] who reported a frequency of 59% of high-risk SE alleles in a predominant black South African female population with RA, and this frequency is similar to that reported by Barnetche *et al*. in a Caucasian population [37]. When considering our results, we cannot exclude that other genetic factors may be involved in Congolese RA patients. This needs further evaluation.

The effect of the environment on the incidence and phenotype of RA must also be considered. Smoking, the main environmental risk factor implicated in RA [38-40], is present only in a small proportion of our patients, and at a low level. Moreover, for cultural reasons women very rarely smoke in DR Congo. Beyond the effect of smoking, other environmental factors could be implicated. Previous studies conducted in sub-Saharan Africa have suggested a protective role of malaria against autoimmune diseases. This applies to the study of Greenwood [41] in Nigeria who observed that mice infected with *Plasmodium falciparum *showed fewer autoimmune disorders than mice free of *P. falciparum*. Similarly, Moolenburgh [42] in Lesotho observed that RA was more frequent and severe in areas of high altitude than on the warmer plains with more malaria. A similar survey in DR Congo could help to clarify this concept, and it remains to be seen whether the tropical environment with its many parasites could interfere with the phenotype of RA or be implicated in the pathogenesis of the disease.

Disease classification, disease activity and functionality of RA were evaluated in this paper using standardized methods that are classical for evaluation of patients seen in the Western world. Unfortunately, mainly because of the cost issue, detailed serology data and genetic typing were not feasible in all patients. Nevertheless, this study will provide a benchmark for measuring benefits of RA care in central Africa and will allow comparing disease patterns in rural compared to this urban population in the future. These preliminary results also make clear that perhaps specific validation is needed for classification criteria outside the Western world. For instance, the very recent new classification criteria for RA [43] are of no use in a specific situation where CRP and CCP measurement is not performed in daily practice and where intensive treatment and a treat-to-target approach are currently unrealistic goals. We also found aspects of timing (duration of morning stiffness, six weeks symptom duration) sometimes problematic to evaluate, as time perceptions are different compared to the Western world. Also proper cross-cultural validation of instruments like the HAQ needs to be carried out, not only because of translation issues, but also because of content problems; for instance, with concepts like 'shampooing hair' which is not often done by Congolese women.

## Conclusion

In conclusion, the current preliminary data on the phenotype and genotype of RA are the first from this part of the world and according to our findings, we put forward the hypothesis that the particular genetic profile of RA in Western Europe is not present in sub-Saharan Africans and this could explain the decreased severity of the disease. A study in a larger sample population is needed to better assess the impact of HLA-DRB1alleles on the prevalence and phenotype of RA in Congolese, and the correlation between the shared epitope and the presence and titers of anti-CCP and RF.

## Abbreviations

ACR: American College of Rheumatology; Anti-CCP antibody: anti-cyclic citrullinated peptide antibody; DAS-28-ESR: Disease Activity Score 28 based on the erythrocyte sedimentation rate; EDTA: ethylene diamine tetra acetic acid; F: female; HAQ: Health Assessment Questionnaire; HILA: Histocompatibility and Immunogenetics Laboratory; HLA: human leucocyte antigen; M: male; OR: odds ratio; PCR: polymerase chain reaction; PPV: positive predictive value; RA: rheumatoid arthritis; RF: rheumatoid factor; SE: shared epitope; SSO: sequence specific oligonucleotide; SSP: sequence specific primer; UHK: University Hospital of Kinshasa

## Competing interests

The authors declare that they have no competing interests.

## Authors' contributions

JJM conceived of the study, participated in its design and coordination, and helped to draft the manuscript, JMM conceived of the study, participated in its design and coordination, and was involved in revising the manuscript. JM performed and scored the X-rays and was involved in revising the manuscript. XB was responsible for the determination of RF on anti-CCP antibodies and helped to draft the manuscript. MPE and KD were responsible for HLA typing and helped to draft the manuscript. RW and PV conceived of the study, participated in its design and coordination, and helped to draft the manuscript. All authors read and approved the final manuscript.
